# Association of Serum Calprotectin and the C-Reactive Protein–Triglyceride–Glucose Index with SYNTAX Score in Patients with Newly Diagnosed Coronary Artery Disease

**DOI:** 10.3390/medicina62050928

**Published:** 2026-05-10

**Authors:** Vahit Demir, Hüseyin Ede, Yaşar Turan, Muhammed Raşid Bakir, Çaglar Alp, Murat Gül, Halil Aktaş, Münire Işlak Demir, Oğuz Yıldırım, Sinan Inci

**Affiliations:** 1 Department of Cardiology, Aksaray Training and Research Hospital, Aksaray 68200, Turkey; oguzyldrm@gmail.com; 2 Clinic of Cardiology, Hamad Medical Corporation Heart Hospital, Doha P.O. Box 3050, Qatar; huseyinede@gmail.com; 3 Department of Cardiology, Samsun University Faculty of Medicine, Samsun 55270, Turkey; yasar044@yahoo.com; 4 Office of Quality Coordination, Aksaray University, Aksaray 68100, Turkey; mrasidbakir@aksaray.edu.tr; 5 Department of Cardiology, Kirikkale University, Kırıkkale 71450, Turkey; drcaglaralp@gmail.com; 6 Department of Cardiology, Aksaray University School of Medicine, Aksaray 68100, Turkey; drmuratgul68@gmail.com (M.G.); halilaktas_85@hotmail.com (H.A.); doktorsinaninci@gmail.com (S.I.); 7 Clinic of Infectious Diseases and Clinical Microbiology, Aksaray Training and Research Hospital, Aksaray 68200, Turkey; dr.mnr@hotmail.com

**Keywords:** coronary artery disease, SYNTAX score, serum calprotectin, C-reactive protein–triglyceride–glucose index, inflammation

## Abstract

*Background and Objectives*: Systemic inflammation is a key driver in the progression and complexity of coronary artery disease (CAD). Serum calprotectin and the C-reactive protein–triglyceride–glucose index (CTI) have emerged as potential inflammatory and metabolic biomarkers; however, their association with angiographic disease severity has not been clearly defined. This study aimed to evaluate the relationship between serum calprotectin, CTI, and the SYNTAX score (SS) in patients with stable CAD. *Materials and Methods*: A total of 134 patients undergoing coronary angiography were enrolled. The SS was calculated to quantify coronary lesion complexity. Patients were classified into two groups based on the results of the coronary angiogram: low SS (n = 73, SS < 23), and intermediate–high SS (n = 61, SS ≥ 23). Serum calprotectin, and CTI were obtained at baseline. Correlation analyses were performed to evaluate associations between biomarkers and SS. Receiver operating characteristic (ROC) curve analysis assessed the ability of these biomarkers to predict intermediate–high SS. Univariable and multivariable logistic regression analyses were performed to determine independent associations. *Results*: Patients with intermediate–high SS had significantly higher levels of serum calprotectin (1009.5 vs. 505.7 ng/mL), and CTI (9.9 vs. 9.5) compared with those with low SS (all *p* < 0.001). Spearman correlation analysis demonstrated significant positive correlations between SS and, serum calprotectin (ρ = 0.488), and CTI (ρ = 0.453) (all *p* < 0.001). ROC analysis showed moderate association in respect to intermediate–high SS (0.739 for serum calprotectin, and 0.722 for CTI). In multivariable models, CTI showed the strongest independent association with intermediate–high SS (OR: 4.66, 95% CI: 2.00–10.84, *p* < 0.001). *Conclusions*: Serum calprotectin and CTI were significantly associated with coronary lesion complexity, as measured by the SS. These biomarkers may serve as valuable tools for identifying patients with greater CAD severity and anatomical complexity.

## 1. Introduction

Coronary artery disease (CAD) is one of the leading causes of morbidity and mortality worldwide [1,2,3]. The anatomical extent and complexity of coronary atherosclerosis are important determinants guiding both revascularization decisions and clinical management [3]. The Synergy between Percutaneous Coronary Intervention and TAXUS and Cardiac Surgery (SYNTAX) score (SS) is a validated angiographic tool designed to measure coronary lesion complexity. It has demonstrated strong prognostic benefit in patients with stable CAD [4]. SS typically ranges from zero to values exceeding 60 in cases of highly complex coronary anatomy. Higher scores reflect a greater anatomical burden and disease complexity [5,6]. According to the SS classification, patients are classified into three groups: low risk (<23), intermediate risk (23–32), and high risk (≥33). It is known that patients with intermediate–high SS have worse clinical outcomes, including increased mortality, myocardial infarction and major adverse cardiovascular events, and generally require more complex revascularization strategies [5]. Regarding prognostic evaluation, findings from the BARI 2D trial indicated that individuals with an intermediate–high SS (≥23) faced a significantly elevated risk for development of major adverse cardiovascular events [7]. Inflammation contributes to all stages of atherosclerosis, including plaque formation, progression, and destabilization. Therefore, systemic inflammatory activity may provide information on disease severity beyond conventional cardiovascular risk factors [8]. C-reactive protein (CRP) is associated with adverse cardiovascular outcomes and increased plaque burden; however, it is a non-specific marker and may not fully represent the inflammatory pathways involved in advanced CAD [9]. Calprotectin (S100A8/A9; MRP8/14) is released mainly by activated neutrophils and monocytes and more directly reflects leukocyte activation and vascular inflammatory activity. Elevated serum calprotectin levels have been associated with endothelial dysfunction, plaque instability, impaired coronary collateral circulation, and adverse cardiovascular outcomes [10,11,12]. Experimental and clinical data also suggest that calprotectin may actively contribute to atherogenesis by promoting leukocyte recruitment and inflammatory signaling within the vascular wall [11]. Metabolic dysregulation is closely linked to inflammation in CAD. The triglyceride–glucose (TyG) index is a marker of insulin resistance and has been associated with cardiovascular risk and atherosclerotic burden [13,14,15]. More recently, composite indices combining inflammatory and metabolic parameters, such as the C-reactive protein–triglyceride–glucose index (CTI), have been proposed to better reflect this inflammatory–metabolic milieu [15]. However, the clinical relevance of CTI and serum calprotectin in relation to coronary lesion complexity remains unclear. Therefore, we aimed to evaluate the association of serum calprotectin and CTI with SS in patients with newly diagnosed CAD.

## 2. Materials and Methods

### 2.1. Study Design and Population

This cross-sectional study was conducted at the Department of Cardiology, Faculty of Medicine, Yozgat Bozok University, Türkiye. The study included 134 consecutive patients aged between 18 and 80 years old who presented with stable angina pectoris or angina-equivalent symptoms and underwent diagnostic coronary angiography between June 2019 and May 2020. Patients were excluded if they met any of the following criteria: acute coronary syndrome; active infection; ongoing inflammatory or autoimmune disorders; prior coronary artery bypass grafting; previous percutaneous coronary intervention; heart failure with a left ventricular ejection fraction (LVEF) < 50%; hypertrophic cardiomyopathy; coronary artery spasm; moderate-to-severe valvular heart disease; congenital cardiac anomalies; thyroid dysfunction; renal impairment (eGFR < 50 mL/min/1.73 m^2^); hepatic dysfunction (transaminase levels exceeding three times the upper normal limit); hematologic disorders; or active malignancy. Additionally, individuals receiving lipid-lowering therapy were excluded to minimize potential effects on metabolic and inflammatory markers. All demographic, clinical, laboratory, and angiographic data were collected prospectively during the index hospitalization.

### 2.2. Clinical Definitions and Echocardiography

Individuals with fasting plasma glucose level of ≥126 mg/dL or receiving oral antidiabetic medication or insulin therapy were considered to have diabetes mellitus. Hypertension was defined as systolic blood pressure of ≥140 mmHg, diastolic blood pressure of ≥90 mmHg, or the use of antihypertensive drugs at the time of evaluation. Hyperlipidemia was defined as a total serum cholesterol level of ≥200 mg/dL. Smoking status was recorded as active tobacco use at the time of clinical assessment. Body mass index (BMI) was calculated by dividing body weight in kilograms by the square of height in meters (kg/m^2^). Transthoracic echocardiography was performed using a Philips Affiniti 50 ultrasound system (Philips Healthcare, Eindhoven, The Netherlands) in accordance with the recommendations of the American Society of Echocardiography. The LVEF was calculated using the modified biplane Simpson’s method.

### 2.3. Laboratory Measurements

Fasting venous blood samples were obtained upon admission, prior to coronary angiography. Samples were collected in 5 mL BD Vacutainer SST II Advance tubes (Becton, Dickinson and Company, Franklin Lakes, NJ, USA) and centrifuged at 1300× *g* for 10 min following clot completion. The separated sera were stored at −80 °C until the time of analysis. Serum calprotectin levels were measured using Human Enzyme-Linked Immunosorbent Assay (ELISA) kits (Sunlong Biotech Co., Ltd., Hangzhou, China), with values expressed in ng/mL. A complete blood count was performed using the XN 1000 analyzer (Sysmex America Inc., Lincolnshire, IL, USA). CRP levels, fasting glucose, triglycerides, and liver and kidney function parameters were measured using the Architect ci4100 automated analyzer (Abbott, Abbott Park, IL, USA). Estimated glomerular filtration rate (eGFR, mL/min/1.73 m^2^) was calculated from serum creatinine, age and sex using the Chronic Kidney Disease Epidemiology Collaboration (CKD-EPI) 2009 equation:

eGFR = 141 × min(Scr/κ, 1)^α^ × max(Scr/κ, 1)^(−1.209)^ × 0.993^Age^ × 1.018 (if female),

where Scr is serum creatinine in mg/dL, κ = 0.7 for females and 0.9 for males, and α = −0.329 for females and −0.411 for males.

The CTI was calculated based on established formulas integrating inflammatory and metabolic components [16], using fasting serum triglyceride, fasting plasma glucose, and CRP levels obtained from venous blood samples collected after an overnight fast of at least 8 h. Triglyceride and glucose values were converted from mg/dL to mmol/L before CTI calculation.

CTI = ln [CRP (mg/L) × triglyceride (mmol/L) × glucose (mmol/L)/2].

### 2.4. Coronary Angiography and Angiographic Evaluation

Coronary angiography was performed via the radial or femoral artery using the standard Judkins technique. Coronary arteries were visualized in cranial, caudal, and right/left oblique projections using a Philips Allura Xper FD10 system (Philips Healthcare, Eindhoven, The Netherlands). Iopromide (Ultravist 370, Schering AG, Berlin, Germany) was used as the contrast medium. Only patients with optimal angiographic image quality were included. Angiograms and quantitative coronary analyses were independently assessed by two experienced cardiologists who were blinded to clinical and laboratory data. Luminal diameter stenosis was recorded according to the American Heart Association reporting system, with the anatomical location and percentage of stenosis documented for each lesion. The SS was calculated for all patients using dedicated software (available at https://syntaxscore.org/calculator/start.htm, accessed on 10 January 2025). Thereafter, the patients were divided into two groups based on the severity of CAD: low SS (n = 73, SS < 23), and intermediate–high SS (n = 61, SS ≥ 23).

### 2.5. Ethical Approval

The study protocol was reviewed and approved by the Local Ethics Committee (Decision Date: 24 April 2019; Approval Number: 2017-KAEK-189-01). The study was conducted in strict accordance with the principles outlined in the Declaration of Helsinki. All procedures involving human participants were performed in compliance with institutional and national ethical standards. All participants were fully informed about the study’s objectives and provided written informed consent prior to enrollment.

### 2.6. Statistical Analysis

All statistical analyses were performed using the Python programming language (version 3.11) with the pandas (2.3.3), NumPy (2.2.6), SciPy (1.15.3), matplotlib (3.10.8), seaborn (0.13.2) and pyreadstat (1.3.3) libraries. The normality of continuous variables was assessed using the Shapiro–Wilk test and further evaluated by visual inspection of histograms and Q–Q plots. As none of the continuous variables showed a normal distribution, data are presented as medians with interquartile ranges (IQR), while categorical variables are expressed as absolute numbers and percentages. Comparisons of continuous variables between groups were performed using the non-parametric Mann–Whitney U test. Associations between inflammatory biomarkers and CAD severity were evaluated using Spearman’s rank correlation analysis. The association degree of CRP, serum calprotectin, and the CTI with presence of intermediate–high SS was assessed using receiver operating characteristic (ROC) curve analysis. Discriminatory ability was quantified by calculating the area under the curve (AUC), and statistical significance was tested against the null hypothesis of an AUC of 0.5. Optimal cut-off values were determined using the Youden index to maximize sensitivity and specificity. Comparisons between ROC curves were conducted using the Hanley and McNeil method [17]. Odds ratios (ORs) with 95% confidence intervals (CIs) were calculated to estimate the risk of intermediate–high coronary complexity associated with elevated biomarker levels. A two-tailed *p*-value of <0.05 was considered statistically significant for all analyses. Supplementary statistical analyses (added during revision). For inclusion in the logistic regression models, sex was coded as a binary indicator (1 = male, 0 = female), and serum calprotectin was rescaled by a factor of 100 so that the corresponding odds ratio represents the change per 100 ng/mL increase. Areas under the receiver operating characteristic curves (AUC) and their 95% confidence intervals were re-computed using the DeLong method, and pairwise differences between correlated ROC curves were tested with the DeLong test. Structural collinearity between CRP and CTI was assessed using variance inflation factors (VIF) and the condition number of the standardised biomarker matrix; CRP and CTI were therefore entered into separate adjusted models. The expanded multivariable logistic regression model was additionally adjusted for body mass index, eGFR (CKD-EPI 2009), LDL-cholesterol and left ventricular ejection fraction. The incremental discriminatory value of calprotectin over CRP was assessed using likelihood-ratio tests, ΔAUC with the DeLong test, the continuous (category-free) Net Reclassification Improvement (NRI) and the Integrated Discrimination Improvement (IDI). To assess possible model overfitting given the moderate sample size, the optimism of each adjusted model’s AUC was estimated by 1000-iteration bootstrap resampling (Harrell).

## 3. Results

### 3.1. Baseline Characteristics of the Study Population

A total of 134 patients with angiographically confirmed CAD were included in the analysis and divided into two groups according to SS: low SS (n = 73) and intermediate–high SS (n = 61). The mean age of the study population was 61.3 ± 7.8 years, and 70.9% of the patients were male. The mean BMI was 24.63 ± 2.2 kg/m^2^, reflecting a predominantly normal-to-overweight population. Common cardiovascular risk factors were highly prevalent, including dyslipidemia (53.7%), diabetes mellitus (51.5%), hypertension (48.5%), and active smoking (42.5%). The mean SS was 24.29 ± 10.99, and 45.5% of patients were classified as having intermediate–high SS. Inflammatory markers demonstrated a mean CRP level of 7.56 ± 5.13 mg/L. Additionally, the mean serum calprotectin level was 838.06 ± 582.03 ng/mL, and the mean CTI was 9.74 ± 0.65. Baseline demographic and clinical characteristics were comparable between the two groups (Table 1). Although patients in the intermediate–high SS group tended to be older, the difference did not reach statistical significance. Systolic and diastolic blood pressure, BMI, heart rate, and LVEF were similar between the groups (all *p* > 0.05). There were no significant differences in smoking status, dyslipidemia, diabetes mellitus, or hypertension. Triglyceride levels were higher in the intermediate–high SS group; however, this difference was borderline and did not reach statistical significance (*p* = 0.053). Routine biochemical parameters did not differ significantly between the groups (all *p* > 0.05). Notably, inflammatory markers showed significant differences. CRP levels were markedly elevated in the intermediate–high SS group compared with the low SS group (9.8 [5.6–15.0] vs. 5.0 [3.0–6.3] mg/L, *p* < 0.001). Similarly, serum calprotectin levels were significantly higher in patients with intermediate–high SS (1009.5 [511.6–1607.6] vs. 505.7 [365.1–684.9] ng/mL, *p* < 0.001). Additionally, the CTI was significantly increased in the intermediate–high SS group (9.9 [9.7–10.2] vs. 9.5 [9.3–9.9], *p* < 0.001).

### 3.2. Correlation Between Inflammatory Biomarkers and SYNTAX Score

**Figure 1:**
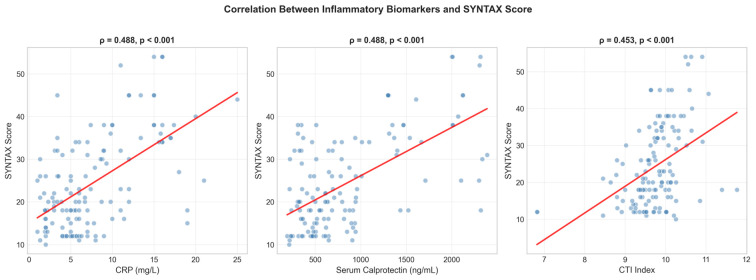
Scatter plots show the correlation between CRP, serum calprotectin, CTI and SYNTAX score. ρ, Spearman rank correlation coefficient. The red line represents the linear regression fit.

Correlation analyses between clinical variables and the SS are presented in Table 2. Spearman correlation analysis demonstrated significant positive associations between the SS and key inflammatory biomarkers. Serum calprotectin showed a moderate correlation with SS (ρ = 0.488, *p* < 0.001), similar to that observed for CRP (ρ = 0.488, *p* < 0.001). CTI was also significantly correlated with SS (ρ = 0.453, *p* < 0.001) (Figure 1). Triglyceride levels showed a weak positive correlation with the SS; however, this relationship was not statistically significant (ρ = 0.168, *p* = 0.053). Furthermore, traditional cardiovascular risk factors and other lipid parameters did not show a significant correlation with the SS. Kendall’s tau-b correlation analysis, performed as a complementary analysis due to the presence of tied ranks in CRP values, confirmed these findings. The Kendall τ-b coefficients were 0.341 for CRP, 0.353 for serum calprotectin, and 0.326 for CTI, all indicating statistically significant associations with the SS (*p* < 0.001 for all).

### 3.3. Comparison According to SYNTAX Score Category

**Figure 2:**
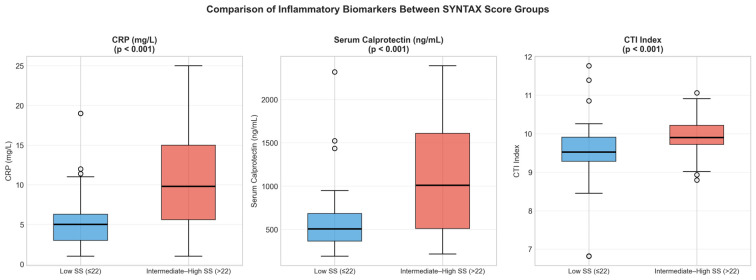
Box plots comparing CRP, serum calprotectin, and CTI between low SYNTAX score (<23) and intermediate–high SYNTAX score (≥23) groups. The central line represents the median; the box represents the interquartile range. *p*-values from Mann–Whitney U test.

When patients were stratified according to SS categories, those with intermediate–high SS exhibited significantly higher median levels of inflammatory biomarkers compared with the low SS group. Median CRP levels were 9.8 mg/L (IQR: 5.6–15.0) in the intermediate–high SS group and 5.0 mg/L (IQR: 3.0–6.3) in the low SS group (*p* < 0.001). Similarly, serum calprotectin levels were significantly elevated in patients with intermediate–high SS (1009.5 ng/mL [IQR: 511.6–1607.7]) compared with those with low SS (505.7 ng/mL [IQR: 365.1–684.9]; *p* < 0.001). CTI was likewise significantly higher in the intermediate–high SS group (median 9.90 vs. 9.52; *p* < 0.001) (Figure 2).

### 3.4. Diagnostic Performance for Intermediate–High SYNTAX Score

**Figure 3:**
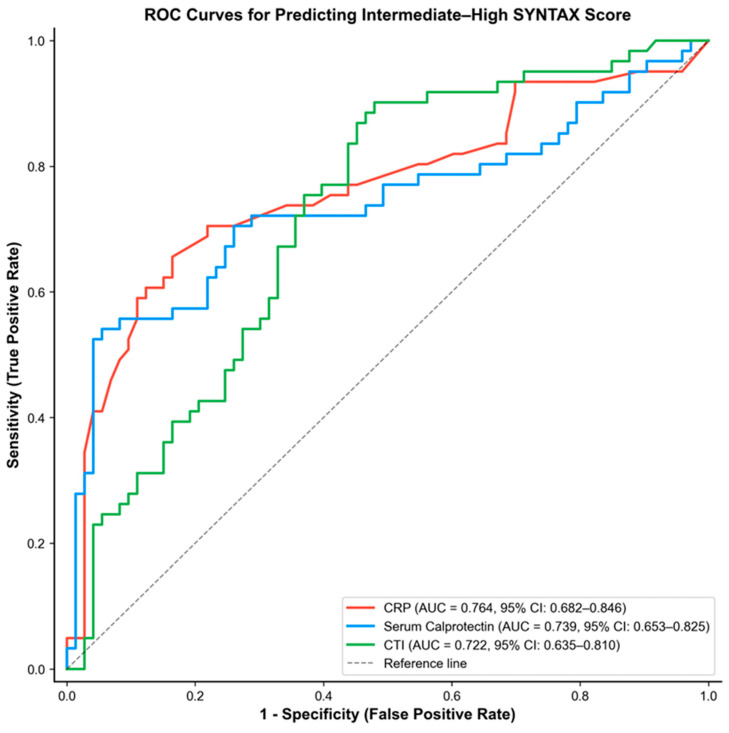
Receiver operating characteristic (ROC) curves comparing the diagnostic performance of CRP, serum calprotectin, and CTI for predicting intermediate–high SYNTAX score (≥23). AUC, area under the curve; CI, confidence interval.

ROC curve analysis was performed to evaluate the association degree of CRP, serum calprotectin, and CTI with intermediate–high SYNTAX score (≥23). In multivariable logistic regression analysis, CTI showed the strongest independent association with this outcome. CRP demonstrated the highest discriminatory performance with an AUC of 0.764 (95% CI: 0.682–0.846, *p* < 0.001), followed by serum calprotectin (AUC: 0.739; 95% CI: 0.653–0.825, *p* < 0.001) and CTI (AUC: 0.722; 95% CI: 0.635–0.810, *p* < 0.001) (Figure 3). Optimal cut-off values derived using the Youden index were 7.40 mg/L for CRP, 944.28 ng/mL for serum calprotectin, and 9.56 for CTI (Table 3).

Pairwise comparisons of the ROC curves were performed using the Hanley–McNeil method to evaluate whether the discriminatory performance of the three biomarkers differed significantly (Table 4). The comparison between CRP and serum calprotectin showed a small difference in AUC that was not statistically significant (ΔAUC = 0.025; z = 0.480; *p* = 0.631). Similarly, the difference between CRP and CTI did not reach statistical significance (ΔAUC = 0.042; z = 0.802; *p* = 0.422). Likewise, serum calprotectin and CTI demonstrated no significant difference in discriminatory performance (ΔAUC = 0.017; z = 0.298; *p* = 0.766).

To further quantify the association between inflammatory biomarkers and coronary lesion complexity, univariable and multivariable logistic regression analyses were performed with intermediate–high SS (≥23) as the dependent variable and continuous biomarker levels as independent variables (Table 5). In the univariable analysis, higher levels of all three biomarkers were significantly associated with an increased likelihood of intermediate–high SS. Each 1 mg/L increase in CRP was associated with 27% higher odds of intermediate–high coronary complexity (OR: 1.27; 95% CI: 1.15–1.40; *p* < 0.001). Similarly, for serum calprotectin, each 100 ng/mL increase corresponded to a 24% increase in odds of intermediate–high SS (OR: 1.24; 95% CI: 1.13–1.35; *p* < 0.001). Among the evaluated biomarkers, CTI demonstrated the strongest association, with each 1-unit increase associated with a 3.82-fold higher odds of intermediate–high coronary complexity (OR: 3.82; 95% CI: 1.79–8.16; *p* < 0.001). After adjustment for potential confounders including age, sex, diabetes mellitus, hypertension, smoking status, BMI and eGFR, the associations remained robust in the multivariable logistic regression analysis. The adjusted OR for CRP remained 1.27 per 1 mg/L increase (95% CI: 1.15–1.41; *p* < 0.001), while serum calprotectin retained a significant association with an adjusted OR of 1.24 per 100 ng/mL increase (95% CI: 1.13–1.36; *p* < 0.001). CTI continued to show the strongest independent association, with an adjusted OR of 4.66 per unit increase (95% CI: 2.00–10.84; *p* < 0.001).

### 3.5. Collinearity and Joint Model Findings

Moderate correlations were observed among CRP, CTI, and calprotectin (r ≈ 0.52–0.54). VIF values (<2) and a low condition number (2.21) indicated no significant multicollinearity. In the joint model, only CRP remained significant (OR: 1.26, *p* < 0.001), whereas CTI was not (Table 6).

### 3.6. Discrimination of Models

Adding CRP to the base clinical model improved AUC from 0.633 to 0.781 (*p* < 0.001), and further inclusion of calprotectin increased AUC to 0.820. The CTI model showed lower discrimination (AUC: 0.744) (Table 7).

### 3.7. Reclassification Analysis

Inclusion of calprotectin into the CRP-based model significantly improved risk reclassification, as shown in Table 8 (NRI: +0.638; IDI: +0.069; both *p* < 0.01).

### 3.8. After Expanded Multivariable Adjustment

After expanded multivariable adjustment for BMI, eGFR (CKD-EPI), LDL-C and LVEF, all three biomarkers remained independently associated with intermediate–high SS (Table 9), and bootstrap internal validation showed only modest optimism (ΔAUC ≤ 0.07 for all three adjusted models).

## 4. Discussion

This study demonstrated a significant association between serum calprotectin, CRP, and CTI and angiographic coronary complexity assessed by SS in patients with stable CAD. Although these biomarkers showed moderate but significant correlations with SS, CTI showed the strongest independent association in multivariable analysis. ROC analysis also showed statistically significant discriminatory ability; however, the AUC values were moderate. Therefore, these biomarkers should be considered supportive, rather than standalone, tools for anatomical risk stratification. Previous studies have reported that elevated CRP levels are associated with adverse cardiovascular outcomes and increased atherosclerotic events. In addition, CRP has been widely used as a marker of systemic inflammation in CAD [8,9]. Our findings suggest that CRP is related not only to clinical outcomes but also to angiographic disease complexity. Calprotectin is concentrated in areas of inflammatory cell infiltration, and increased expression has been linked to intraplaque inflammation and tissue destruction, suggesting a role as a biomarker in acute coronary syndrome [11]. This is consistent with evidence linking calprotectin to plaque vulnerability and adverse cardiovascular outcomes. Demir et al. reported that higher serum calprotectin levels were independently associated with impaired coronary collateral circulation in patients with stable CAD and suggested a relationship between inflammatory activity and adverse coronary vascular remodeling [12]. These findings support the concept that calprotectin reflects not only systemic inflammation but also localized inflammatory processes that may influence coronary anatomy and microvascular function. Such mechanisms may explain the association between elevated serum calprotectin levels and greater angiographic coronary complexity observed in our study. Serum calprotectin has also been associated with adverse outcomes in other ischemic vascular conditions. Higher concentrations have been linked to greater disease severity and worse functional outcomes in acute ischemic events, suggesting that calprotectin reflects an inflammatory response that contributes to tissue injury and poor prognosis [18]. In addition, higher serum calprotectin levels have been observed in overweight individuals and those with impaired glucose metabolism, even in apparently healthy populations, suggesting that inflammatory activation may precede clinical manifestations of CAD [19,20]. Overall, these data support calprotectin as a biomarker with prognostic relevance across vascular beds. In our study, serum calprotectin showed moderate discrimination between moderate and high SS (AUC: 0.739; 95% CI: 0.653–0.825). Sensitivity was 54.1% and specificity was 94.5%, indicating that higher calprotectin levels may identify patients with advanced coronary lesion complexity with high specificity. In a cohort of 1007 patients with acute coronary syndrome, Xiong et al. showed that the TyG index independently predicted intermediate–high coronary complexity defined as SS ≥ 23. They reported a weak but significant correlation between the TyG index and SS (r = 0.22, *p* < 0.001), and TyG remained an independent predictor in multivariable logistic regression (OR: 2.645; 95% CI: 1.902–3.679; *p* < 0.001), with an AUC of 0.631 (95% CI: 0.588–0.674) [21]. These results support the role of metabolic dysregulation in coronary lesion complexity. CTI integrates systemic inflammation and metabolic dysfunction, with CRP also contributing to the TyG index, and was significantly associated with coronary lesion complexity in our study, consistent with the findings above. ROC analysis showed that CTI predicted intermediate–high SS with an AUC of 0.722 (95% CI: 0.635–0.810), indicating moderate discrimination. The optimal cut-off value of 9.56 yielded a sensitivity of 90.2%, suggesting potential value for identifying patients at increased anatomical risk. In multivariable logistic regression analysis, CTI remained an independent predictor of intermediate–high SS; each one-unit increase was associated with 4.66-fold higher odds of greater coronary complexity (95% CI: 2.00–10.84, *p* < 0.001). Thus, CTI may provide complementary information beyond traditional metabolic markers when identifying patients with more complex CAD. Recent studies have further supported CTI as a marker of extensive coronary involvement. Machine learning-based analyses have shown that CTI predicts multivessel disease and may outperform several traditional cardiovascular risk factors in identifying advanced coronary pathology [22]. In line with this perspective, classical risk factors in our cohort were not significantly associated with SS. Evidence from other cardiovascular populations, including patients with heart failure [23], elderly individuals [24], and those undergoing percutaneous coronary intervention, also supports the prognostic value of indices integrating inflammation and insulin resistance [25]. Together, these studies indicate that CRP–triglyceride–glucose-based indices link metabolic dysfunction and inflammatory activity with angiographic coronary complexity and adverse outcomes. Because CTI incorporates CRP in its formula, the similarity in predictive performance between CTI and CRP is expected to some extent. Two methodological points warrant explicit comment. First, because CTI is defined as ln[CRP × triglyceride × glucose/2] it shares structural variance with CRP. In our cohort the two markers were strongly correlated (Spearman ρ = 0.63); the variance inflation factors confirmed moderate but not severe collinearity when each marker was regressed on the other two (CRP 1.73, calprotectin 1.38, CTI 1.43; condition number 2.21). When CRP and CTI were entered jointly into the same multivariable model, however, the CTI effect collapsed (OR 1.42, *p* = 0.480) while CRP retained its effect (OR 1.26, *p* = <0.001), empirically confirming that the CTI signal in this cohort is largely CRP-driven. Accordingly, CRP and CTI are reported in separate adjusted models and their adjusted odds ratios should not be directly compared. Second, the incremental discriminatory value of calprotectin over CRP was modest. Adding calprotectin to a base + CRP model improved fit by the likelihood-ratio test (χ^2^ = 11.33, *p* < 0.001) and yielded a statistically significant continuous Net Reclassification Improvement (+0.638, *p* < 0.001) and Integrated Discrimination Improvement (+0.0692, *p* = 0.003); however, the corresponding ΔAUC was small and not statistically significant by the DeLong test. Calprotectin therefore appears to add reclassification information beyond CRP without substantially increasing overall discrimination, and the three biomarkers should be considered complementary rather than competing markers of the inflammatory milieu underlying complex coronary anatomy. Future studies should address potential collinearity and clarify the independent contribution of each biomarker using appropriate statistical approaches. This study has several limitations. First, it is a single-center study with a relatively small sample size. Second, the cross-sectional design limits causal inference; prospective studies are needed to determine whether serum calprotectin or CTI contributes directly to the severity of coronary atherosclerosis. Third, biomarker measurements were obtained only once, and temporal changes in inflammatory or metabolic status were not assessed. Finally, residual confounding cannot be excluded. Information on medication intensity, inflammatory comorbidities, and lifestyle factors was limited and may have influenced biomarker levels.

## 5. Conclusions

In patients with stable CAD, serum calprotectin and CTI were significantly associated with angiographic coronary lesion complexity, as assessed by SS. Both biomarkers showed moderate but significant correlations with SS and moderate discriminatory performance for identifying patients with moderate-to-high anatomical disease burden. Larger, population-based studies are needed to better define the clinical utility of these biomarkers and their potential role in risk stratification.

## Figures and Tables

**Table 1:** Baseline demographic and clinical characteristics of patients according to SYNTAX score category.

Variable	Low SS (<23) (n = 73)	Intermediate–High SS (≥23) (n = 61)	*p*-Value
Age (years)	60 (55–67)	65 (57–67)	0.076
Body Mass Index (kg/m^2^)	24.5 (23.5–26.5)	24.1 (23.4–25.8)	0.252
Systolic BP (mmHg)	130 (130–140)	135.0 (127.5–140.0)	0.286
Diastolic BP (mmHg)	80 (78–80)	80 (75–80)	0.960
Heart Rate (bpm)	78 (76–80)	78 (75–80)	0.454
Fasting Glucose (mg/dL)	100 (90–132)	98 (90–123)	0.321
BUN (mg/dL)	25 (23–30)	25 (21–28)	0.278
Creatinine (mg/dL)	0.9 (0.8–1.0)	0.8 (0.8–0.9)	0.286
eGFR (mL/min/1.73 m^2^)	88.5 (79.2–99.2)	91.1 (78.4–96.8)	0.801
Total Cholesterol (mg/dL)	213 (191–234)	219 (209–234)	0.177
Triglycerides (mg/dL)	145 (114–183)	166 (140–193)	0.053
HDL-C (mg/dL)	40 (37–45)	40 (34–41)	0.273
LDL-C (mg/dL)	124 (110–145)	132 (118–145)	0.161
AST (U/L)	18 (14–20)	18 (11–21)	0.547
ALT (U/L)	22 (18–26)	21 (18–25)	0.885
CRP (mg/L)	5.0 (3.0–6.3)	9.8 (5.6–15.0)	**<0.001**
WBC (×10^3^/µL)	7.8 (7.0–8.9)	7.8 (6.7–8.9)	0.845
Hemoglobin (g/dL)	14.4 (13.3–15.3)	14.6 (13.8–15.8)	0.505
Platelet (×10^3^/µL)	250 (201–275)	250 (172–276)	0.591
Serum Calprotectin (ng/mL)	505.7 (365.1–684.9)	1009.5 (511.6–1607.6)	**<0.001**
CTI	9.5 (9.3–9.9)	9.9 (9.7–10.2)	**<0.001**
LVEF (%)	52 (50–54)	53 (52–54)	0.431
Gender			0.773
Male, n (%)	51 (69.9)	44 (72.1)	
Female, n (%)	22 (30.1)	17 (27.9)	
Smoking Status			0.494
Non-smoker, n (%)	40 (54.8)	37 (60.7)	
Smoker, n (%)	33 (45.2)	24 (39.3)	
Dyslipidemia			0.938
Absent, n (%)	34 (46.6)	28 (45.9)	
Present, n (%)	39 (53.4)	33 (54.1)	
Diabetes Mellitus			0.887
Absent, n (%)	35 (47.9)	30 (49.2)	
Present, n (%)	38 (52.1)	31 (50.8)	
Hypertension			0.213
Absent, n (%)	34 (46.6)	35 (57.4)	
Present, n (%)	39 (53.4)	26 (42.6)	

Data are presented as median (interquartile range) for continuous variables and n (%) for categorical variables. *p*-values from Mann–Whitney U test for continuous variables and chi-square test for categorical variables. Bold *p*-values indicate statistical significance (*p* < 0.05). SS, SYNTAX score; BP, blood pressure; BUN, blood urea nitrogen; eGFR, estimated glomerular filtration rate; HDL-C, high-density lipoprotein cholesterol; LDL-C, low-density lipoprotein cholesterol; AST, aspartate aminotransferase; ALT, alanine aminotransferase; CRP, C-reactive protein; WBC, white blood cell; CTI, CRP–triglyceride–glucose index; LVEF, left ventricular ejection fraction.

**Table 2:** Correlation coefficients between clinical variables and SYNTAX score.

Variable	Spearman ρ	Kendall τ-b	*p*-Value
CRP (mg/L)	0.488	0.341	**<0.001**
Serum Calprotectin (ng/mL)	0.488	0.353	**<0.001**
CTI	0.453	0.326	**<0.001**
Triglycerides (mg/dL)	0.168	—	0.053
Body Mass Index (kg/m^2^)	−0.139	—	0.110
Total Cholesterol (mg/dL)	0.130	—	0.133
Hemoglobin (g/dL)	0.121	—	0.169
LDL-C (mg/dL)	0.103	—	0.238
HDL-C (mg/dL)	−0.090	—	0.303
Age (years)	0.085	—	0.327
AST (U/L)	−0.083	—	0.339
WBC (×10^3^/µL)	−0.083	—	0.347
Heart Rate (bpm)	0.078	—	0.372
Systolic BP (mmHg)	0.073	—	0.401
BUN (mg/dL)	−0.073	—	0.405
ALT (U/L)	−0.062	—	0.474
Creatinine (mg/dL)	−0.055	—	0.530
LVEF (%)	0.046	—	0.596
Platelet (×10^3^/µL)	0.030	—	0.737
Diastolic BP (mmHg)	0.021	—	0.809
Fasting Glucose (mg/dL)	−0.019	—	0.826

ρ, Spearman rank correlation coefficient; τ-b, Kendall’s tau-b rank correlation coefficient. Kendall’s tau-b values are presented for the three key biomarkers as a complementary analysis given the substantial number of tied ranks in CRP values (56 unique values among 134 observations). *p*-values correspond to the Spearman correlation. Bold *p*-values indicate statistical significance (*p* < 0.05). CRP and serum calprotectin yielded nearly identical Spearman coefficients at three decimal places; at five decimal precision: CRP ρ = 0.48834, serum calprotectin ρ = 0.48831. CRP, C-reactive protein; CTI, CRP–triglyceride–glucose index.

**Table 3:** Receiver operating characteristic curve analysis for predicting intermediate–high SYNTAX score.

Variable	AUC (95% CI)	*p*-Value	Cut-Off	Sensitivity (%)	Specificity (%)	Youden Index
CRP	0.764 (0.682–0.846)	**<0.001**	7.40	65.6	83.6	0.491
Serum Calprotectin	0.739 (0.653–0.825)	**<0.001**	944.28	54.1	94.5	0.486
CTI	0.722 (0.635–0.810)	**<0.001**	9.56	90.2	52.1	0.422

AUC, area under the curve; CI, confidence interval. Optimal cut-off values determined by the Youden index. *p*-values represent comparison against the null hypothesis (AUC = 0.5) using the Hanley–McNeil method. CRP, C-reactive protein; CTI, CRP–triglyceride–glucose index. Bold *p*-values indicate statistical significance (*p* < 0.05).

**Table 4:** Pairwise comparison of ROC curves using the Hanley–McNeil method.

Comparison	ΔAUC	z-Statistic	*p*-Value
CRP vs. Serum Calprotectin	0.025	0.480	0.631
CRP vs. CTI	0.042	0.802	0.422
Serum Calprotectin vs. CTI	0.017	0.298	0.766

ΔAUC, difference in area under the curve. *p*-values from Hanley–McNeil method for comparing correlated ROC curves.

**Table 5:** Univariable and multivariable logistic regression analysis for predicting intermediate–high SYNTAX score.

Biomarker	Unadjusted OR (95% CI)	*p*-Value	Adjusted OR * (95% CI)	*p*-Value
CRP (per 1 mg/L)	1.27 (1.15–1.40)	**<0.001**	1.27 (1.15–1.41)	**<0.001**
Serum Calprotectin (per 100 ng/mL)	1.24 (1.13–1.35)	**<0.001**	1.24 (1.13–1.36)	**<0.001**
CTI (per 1 unit)	3.82 (1.79–8.16)	**<0.001**	4.66 (2.00–10.84)	**<0.001**

OR, odds ratio; CI, confidence interval. Odds ratios represent the risk per unit increase in each biomarker. Serum calprotectin OR is expressed per 100 ng/mL increase. * Adjusted for age, sex, diabetes mellitus, hypertension, and smoking status. CRP, C-reactive protein; CTI, CRP–triglyceride–glucose index. Bold *p*-values indicate statistical significance (*p* < 0.05).

**Table 6:** Collinearity diagnostics for CRP, calprotectin and CTI; effect of joint entry of CRP and CTI in the multivariable model.

Statistic	Value	Statistic	Value
Pearson r (CRP vs. CTI)	0.544	Spearman ρ (CRP vs. CTI)	0.630
Pearson r (CRP vs. Calprotectin)	0.521	Spearman ρ (CRP vs. Calprotectin)	0.398
VIF—CRP	1.73	Tolerance—CRP	0.577
VIF—Calprotectin	1.38	Tolerance—Calprotectin	0.724
VIF—CTI	1.43	Tolerance—CTI	0.699
Condition number	2.21	—	—
Joint model: CRP OR (95% CI), *p*	1.26 (1.11–1.42), <0.001	Joint model: CTI OR (95% CI), *p*	1.42 (0.54–3.76), 0.480

VIF, variance inflation factor. The joint model is adjusted for age, sex, diabetes mellitus, hypertension, smoking, BMI, eGFR (CKD-EPI), LDL-C and LVEF. CRP and CTI are reported in separate adjusted models in the main analysis because of structural collinearity (CTI is mathematically derived from CRP).

**Table 7:** Discrimination of nested logistic models for intermediate–high SYNTAX score (≥23).

Model	Predictors	AUC	95% CI (DeLong)	LR χ^2^ vs. Prior	*p*
Base (clinical)	Age, sex, DM, HT, smoking	0.633	(0.538–0.728)	—	—
Base + CRP	+CRP	0.781	(0.701–0.862)	32.30 (df 1)	<0.001
Base + CRP + Calprotectin	+calprotectin (per 100 ng/mL)	0.820	(0.746–0.895)	11.33 (df 1)	<0.001
Base + CTI	+CTI (CRP not entered)	0.744	(0.661–0.827)	17.33 (df 1)	<0.001

Complete-case sample n = 134, events = 61. Base model: age, sex, diabetes mellitus, hypertension, smoking. AUC, area under the curve; CI, confidence interval (DeLong); LR, likelihood-ratio test against the immediately preceding (nested) model.

**Table 8:** Continuous Net Reclassification Improvement (NRI) and Integrated Discrimination Improvement (IDI) for the addition of calprotectin to a model containing CRP.

Metric	Estimate	95% CI	*p*
NRI (continuous, category-free)	+0.638	(+0.316 to +0.959)	<0.001
IDI	+0.0692	(+0.0242 to +0.1141)	0.003

Reference model: base clinical covariates + CRP. Test model: reference model + serum calprotectin (per 100 ng/mL). NRI uses the continuous (category-free) formulation of Pencina; IDI is the Pencina Integrated Discrimination Improvement.

**Table 9:** Expanded multivariable logistic regression for intermediate–high SYNTAX score (≥23) with bootstrap-corrected discrimination.

Biomarker	Adjusted OR	95% CI	*p*	Apparent AUC	Optimism	Corrected AUC
CRP (per 1 mg/L).	1.29	(1.16–1.43)	<0.001	0.807	+0.057	0.750
Serum calprotectin (per 100 ng/mL)	1.28	(1.15–1.43)	<0.001	0.793	+0.065	0.728
CTI (per 1 unit)	4.67	(1.99–10.95)	<0.001	0.758	+0.074	0.684

Each biomarker was entered separately into a multivariable model adjusted for age, sex, diabetes mellitus, hypertension, smoking, BMI, eGFR (CKD-EPI 2009), LDL-cholesterol and LVEF (complete-case n = 134). Optimism was estimated by 1000 bootstrap resamples (Harrell). OR, odds ratio.

## Data Availability

The data presented in this study are available on request from the corresponding author. The data are not publicly available due to ethical restrictions and patient privacy.

## Abbreviations

The following abbreviations are used in this manuscript:

AUC	Area under the curve
BMI	Body mass index
CAD	Coronary artery disease
CI	Confidence interval
CRP	C-reactive protein
CTI	C-reactive protein–triglyceride–glucose index
ELISA	Enzyme-linked immunosorbent assay
IQR	Interquartile range
LVEF	Left ventricular ejection fraction
OR	Odds ratio
ROC	Receiver operating characteristic
SS	SYNTAX score
TyG	Triglyceride–glucose index

## References

[1] Kodeboina M., Piayda K., Jenniskens I., Vyas P., Chen S., Pesigan R.J., Ferko N., Patel B.P., Dobrin A., Habib J. (2023). Challenges and Burdens in the Coronary Artery Disease Care Pathway for Patients Undergoing Percutaneous Coronary Intervention: A Contemporary Narrative Review. Int. J. Environ. Res. Public Health.

[2] Sultana N., Ha F.J., White A., Brown A.J., Nerlekar N. (2025). Revascularization in Stable Coronary Disease: A Systematic Review and Meta-Analysis of Randomized Clinical Trials. Int. J. Angiol..

[3] Iqbal J., Serruys P.W., Taggart D.P. (2013). Optimal revascularization for complex coronary artery disease. Nat. Rev. Cardiol..

[4] Banning A.P., Serruys P., De Maria G.L., Ryan N., Walsh S., Gonzalo N., van Geuns R.J., Onuma Y., Sabate M., Davies J. (2022). Five-year outcomes after state-of-the-art percutaneous coronary revascularization in patients with de novo three-vessel disease: Final results of the SYNTAX II study. Eur. Heart J..

[5] Byrne R.A., Fremes S., Capodanno D., Czerny M., Doenst T., Emberson J.R., Falk V., Gaudino M., McMurray J.J.V., Mehran R. (2023). 2022 Joint ESC/EACTS review of the 2018 guideline recommendations on the revascularization of left main coronary artery disease in patients at low surgical risk and anatomy suitable for PCI or CABG. Eur. Heart J..

[6] Masuda S., Serruys P.W., Kageyama S., Kotoku N., Ninomiya K., Garg S., Soo A., Morel M.A., Puskas J.D., Narula J. (2023). Treatment recommendation based on SYNTAX score 2020 derived from coronary computed tomography angiography and invasive coronary angiography. Int. J. Cardiovasc. Imaging.

[7] Ikeno F., Brooks M.M., Nakagawa K., Kim M.K., Kaneda H., Mitsutake Y., Vlachos H.A., Schwartz L., Frye R.L., Kelsey S.F. (2017). SYNTAX score and long-term outcomes: The BARI-2D trial. J. Am. Coll. Cardiol..

[8] Libby P. (2002). Inflammation in atherosclerosis. Nature.

[9] Ridker P.M. (2007). C-reactive protein and the prediction of cardiovascular events among those at intermediate risk: Moving an inflammatory hypothesis toward consensus. J. Am. Coll. Cardiol..

[10] Sun Y., Xu H., Gao W., Deng J., Song X., Li J., Liu X. (2024). S100A8/A9 proteins: Critical regulators of inflammation in cardiovascular diseases. Front. Cardiovasc. Med..

[11] Zuo Y., NaveenKumar S.K., Navaz S., Liang W., Sugur K., Kmetova K., Ayers C.R., Kluge L., Chong E., Shah A.M. (2025). Epidemiological and translational study of calprotectin and atherosclerotic cardiovascular disease. JAMA Cardiol..

[12] Demir V., Ede H., Ercan M., Turan Y., Hidayet S., Inandiklioglu N., Erbay A.R. (2019). Relationship of serum calprotectin, angiopoietin 1, and angiopoietin 2 levels with coronary collateral circulation in patients with stable coronary artery disease. Kardiol. Pol..

[13] Sun Y., Guo Y., Ma S., Mao Z., Meng D., Xuan K., Lu R., Pan X., Zhu X. (2025). Association of C-reactive protein–triglyceride glucose index with the incidence and mortality of cardiovascular disease: A retrospective cohort study. Cardiovasc. Diabetol..

[14] Lu Z., Li L., Wang X., Lv L., Rong S., Li B. (2025). Association between C-reactive protein–triglyceride glucose index and future cardiovascular disease risk in a population with cardiovascular–kidney–metabolic syndrome stage 0–3. Sci. Rep..

[15] Chen Y., Jia W., Guo J., Yang H., Sheng X., Wei L., Li J. (2025). Association between the C-reactive protein–triglyceride glucose index and new-onset coronary heart disease among metabolically heterogeneous individuals. Cardiovasc. Diabetol..

[16] Ruan G.T., Xie H.L., Zhang H.Y., Liu C.A., Ge Y.Z., Zhang Q., Wang Z.W., Zhang X., Tang M., Song M.M. (2022). A novel inflammation and insulin resistance related indicator to predict the survival of patients with cancer. Front. Endocrinol..

[17] Hanley J.A., McNeil B.J. (1982). The meaning and use of the area under a receiver operating characteristic (ROC) curve. Radiology.

[18] Li H., Zhao K., Yu W. (2025). Correlation of serum calprotectin with outcome of acute cerebral infarction. Open Med..

[19] Bergmann K., Stefańska A., Kuligowska-Prusińska M., Krintus M. (2025). Serum calprotectin is associated with overweight and laboratory markers of glucose metabolism in apparently healthy young adults—A cross-sectional descriptive study. Metabolites.

[20] Cozac D.-A., Halațiu V.-B., Scridon A. (2025). The alarmin tandem: Unraveling the complex effect of S100A8/A9—From atherosclerosis to cardiac arrhythmias. Front. Immunol..

[21] Xiong S., Chen Q., Long Y., Su H., Luo Y., Liu H., Chen Y., Feng Q., Peng X., Jiang M. (2023). Association of the triglyceride–glucose index with coronary artery disease complexity in patients with acute coronary syndrome. Cardiovasc. Diabetol..

[22] Hou L., Li Y., Liu Q. (2025). C-reactive protein–triglyceride glucose index in predicting three-vessel coronary artery disease risk: A retrospective study using machine learning approaches. Ann. Med..

[23] Kaya Ç., Öztürk C. (2026). Prognostic value of the CRP-TyG index for in-hospital and 1-year mortality in patients with decompensated heart failure. BMC Cardiovasc. Disord..

[24] Ni G., Chen Z., Zhu A., Cheang I., Zhu X., Fu Y., Zhang H., Li X. (2026). Association between C-reactive protein–triglyceride glucose index (CTI) and cardiovascular and all-cause mortality risk among the elderly population: Insights from three datasets. Clin. Res. Cardiol..

[25] Gao A., Peng B., Gao Y., Yang Z., Li Z., Guo T., Qiu H., Gao R. (2025). Evaluation and comparison of inflammatory and insulin resistance indicators on recurrent cardiovascular events in patients undergoing percutaneous coronary intervention: A single-center retrospective observational study. Diabetol. Metab. Syndr..

